# Lymphatic mapping Of Oropharyngeal Cancer (LOOC): protocol for a phase II surgical imaging trial to evaluate contralateral drainage and occult metastasis in oropharyngeal cancer

**DOI:** 10.1136/bmjopen-2025-101746

**Published:** 2025-06-05

**Authors:** Clare Schilling, Karl Payne, Simon Wan, Chris Brew-Graves, Enyi Ofo, Gareth Ambler, Alex Weller, Gopinath Gnanasegaran, Vinidh Paleri, Richard Shaw, Jason Fleming, David Walker, Mandeep Bajwa, Mark McGurk

**Affiliations:** 1 Department of Head and neck Surgery, University College Hospital, London, UK; 2 Head and Neck Academic Centre, University College, London, UK; 3 Institute of Nuclear Medicine, University College London/University College London Hospitals NHS Foundation Trust, London, UK; 4 Centre for Medical Imaging, Division of Medicine, University College, London, UK; 5 Department of ENT Surgery, St Georges Hospital NHS Foundation Trust, London, UK; 6 Department of Statistical Science, University College, London, UK; 7 Department of Radiology, St Georges Hospital NHS Foundation Trust, London, UK; 8 Department of Nuclear Medicine, Royal Free Hospital NHS Foundation Trust, London, UK; 9 Head and Neck Unit, Royal Marsden Hospital NHS Foundation Trust, London, UK; 10 Department of Molecular and Clinical Cancer Medicine, Liverpool University, Liverpool, UK; 11 Department of Head and Neck Surgery, Aintree University Hospital, Liverpool, UK; 12 Department of ENT Surgery, Royal Surrey NHS Foundation Trust, Cranleigh, UK; 13 Department of Oral and Maxillofacial Surgery, Royal Surrey NHS Foundation Trust, Cranleigh, UK; 14 Section of Oncology, University of Surrey, Guildford, UK

**Keywords:** patients, head & neck surgery, head & neck imaging, nuclear radiology

## Abstract

**Introduction:**

Treatment of the node negative contralateral neck in oropharyngeal cancer (OPC) remains debated, with no clear consensus. Prophylactic contralateral neck treatment (either surgically or via irradiation) is generally recommended when the estimated risk of occult nodal metastasis is >20%. Unfortunately, patients undergoing bilateral neck treatment often require long-term supportive care for swallowing dysfunction. Reducing the impact of treatment on long-term quality of life is key in patients with OPC who have a good prognosis and tend to be young and fit at presentation. Lymphatic mapping and the use of free-hand single photon emission CT (fhSPECT) combined with sentinel lymph node biopsy is a novel approach to address this clinical need. The Lymphatic mapping Of Oropharyngeal Cancer trial aims to (a) validate a lymphatic mapping protocol in OPC using new technology (fhSPECT) with radiotracers and (b) establish lymphatic drainage patterns and the occult metastatic rate in the contralateral neck in OPC.

**Methods and analysis:**

The design is a prospective multicentre cohort trial to understand the lymphatic drainage pattern in 150 patients with OPC and unilateral neck metastases. The trial has two phases: (1) *imaging phase (n=75)*—aim: develop an imaging protocol to establish the lymphatic drainage pattern in a population of patients with proven unilateral neck metastasis from OPC. The intervention will involve peritumoural injection of radiotracer followed by fhSPECT scan under general anaesthesia (GA) (at time of examination under anaesthetic). A SPECT/CT scan (gold standard for lymphatic mapping) will be carried out subsequently as a comparator. The primary outcome is the rate of contralateral drainage. Secondary outcome is the accuracy of fhSPECT versus SPECT/CT. The number of contralateral nodes on SPECT/CT will be used as the denominator in calculating the sensitivity of fhSPECT in independently verified images. fhSPECT should achieve sensitivity >94%. A minimum number of 20/75 patients will be required to demonstrate contralateral drainage to proceed to the surgical stage. An imaging substudy (n=20) aims to develop a secondary imaging protocol in the event of <94% sensitivity of intraoperative fhSPECT. To investigate the sensitivity of outpatient imaging (single injection of radiotracer and SPECT/CT) compared with gold standard (SPECT/CT from initial imaging phase) and the acceptability of outpatient injection compared with under GA. Twenty patients from the imaging phase with easily accessible tumours will be invited to undergo a second imaging; (2) *Surgical phase (n=75)*—aim: demonstrate the utility of surgically staging the contralateral neck using sentinel node biopsy (SNB). The primary outcome of this surgical phase is the occult metastatic rate of contralateral nodes (positive SNB). The contralateral drainage rate will be identified during the imaging phase, with an expected SNB positive rate of excised nodes ranging from 25% to 40%.

**Ethics and dissemination:**

The outcome of this trial will provide a validated protocol and evidence to inform the design of future research in which management of the contralateral neck is based on surgical staging. Ethical approval was granted by the Yorkshire & The Humber-South Yorkshire Research Ethics Committee (REC ref: 20/YH/0111). Results from the trial will be presented to the scientific community at appropriate meetings and international journals. Patients and the public will be informed via patient groups, cancer charities and social media/press releases.

**Trial registration number:**

NCT04498221.

**STRENGTHS AND LIMITATIONS OF THIS STUDY**

- Trial testing new technology to inform patient-specific treatment in a rapidly increasing tumour group (oropharyngeal cancer).
- Novel design incorporating in-built protocol development informs methodology of later stages of the trial.
- Interventions are specifically designed to fit into the current clinical pathway, paving the way for adoption in phase III trials.
- Reliance on technology may limit applicability in resource-poor settings.

## Introduction

### Background and rationale

#### Clinical need

 The Lymphatic mapping Of Oropharyngeal Cancer (LOOC) trial was developed to address patient concerns and published evidence showing an increasing unmet need in the treatment of patients with oropharyngeal cancer (OPC).^1^,^5^ Like other head and neck cancer subsites, OPC spreads to regional lymph nodes (LN) in the neck. Studies show that there is no consensus on the optimal treatment for the clinically node-negative contralateral neck in patients presenting with an OPC and unilateral neck metastasis. Most evidence is based on case series comparing the outcomes of unilateral versus bilateral treatment, with significant heterogeneity of the modality used (conventional/robotic surgery, conformal radiation or intensity-modulated radiation).^6^,^8^ However, all these studies show that morbidity (ie, dysphagia, feeding tube dependency, taste, xerostomia and neck lymphoedema) is significantly improved when only one side of the neck is treated.^9^,^12^ The reason for upfront treatment of the contralateral neck is to control occult metastases that are not detectable by routine staging imaging.

LOOC is an early phase oncology imaging trial, which will evaluate the utility of new technology to detect occult metastasis, thus accurately staging the contralateral neck. The results can then be rapidly translated to late phase prospective clinical trials in which treatment decisions can be based on the outcome of the validated protocol developed in LOOC.

#### Improving health outcomes in the National Health Service

Treatment of OPC is associated with significant morbidity. Traditionally, prophylactic treatment of the contralateral clinically node-negative neck is undertaken when the estimated risk of involvement is >20%.^13^ Patients undergoing bilateral neck treatment often require long-term supportive care for swallowing dysfunction because of changes to the swallowing-related organs (pharyngeal muscles, larynx, salivary glands and oesophagus).^11 12 14 15^ Dependence on feeding via gastrostomy or very limited oral diet is common. Reducing the impact of treatment on long-term function is key in patients with OPC who, in the case of human papillomavirus (HPV)-positive disease, have a good prognosis and tend to be younger and fit at presentation.^10 16^

Unilateral neck treatment has a clear benefit in protecting swallow function and thus improving quality of life. This trial is the first step in developing a technique to accurately stage the contralateral neck and thus spare the majority of patients from undergoing unnecessary treatment to the unaffected neck. If successful, this has major implications for improving survivorship for these patients, who are increasing in number with rising incidence of the disease. The impact for the National Health Service (NHS) will be immediate and long-lasting. Surgical time and inpatient stay will be reduced, as will acute admissions for dehydration during radiotherapy. Fewer patients will require percutaneous feeding, thus reducing complications. The impact on allied health professionals—speech and language therapists and dietitians including provision of dietary supplements will also be reduced. Furthermore, by developing a reliable imaging protocol for lymphatic mapping of deep tumours in LOOC, there will be easy translation to other head and neck cancers such as salivary gland, thyroid and larynx. There may also be translation to other deep body cavity tumours such as the lung and prostate gland, where there is controversy about the exact extent of nodal resection required, and nodal relapse is a major cause of treatment failure.

#### Justification for trial and comparators

The mechanism of reliability of lymphatic mapping to sentinel nodes was first described in 2007.^17^ Functional lymphatic imaging studies were undertaken in tumour and control footpads of mice. Tracer showed in the sentinel node within 2 min of injection compared with 30 min for the control. Histologically, sentinel nodes had c-Myc oncogene overexpression stimulating VEGF-C and VEGF-D to induce lymphangiogenesis in the node, causing a 23-fold increase in lymphatic tracer drainage. Lymphatic mapping reveals an active premetastatic process directing lymphatic flow to specific nodes. Human studies in oral cancer showed patients injected and imaged on two separate occasions a week apart had drainage to identical nodes.^18^

The LOOC trial will allow a choice of lymphatic mapping radiotracer, 99mTc-human albumin colloidal particles or 99mTc-Lymphoseek. 99mTc-human albumin colloidal particles are standard radiotracers, which accumulate within LN. These have a large body of evidence supporting efficacy in the mapping of sentinel nodes from oral cancer and more recently have been used to map lymphatic drainage in patients with cN0 OPC under general anaesthetic.^19^ In this study, patients were imaged by single photon emission CT (SPECT)/CT 3–6 hours postinjection with a contralateral drainage rate of 20%.

Lymphoseek has additional properties to aid retention in the sentinel node via binding to macrophage CD206 mannose receptor. The advantage of rapidly clearing the injection site while selectively retaining the substance in the sentinel node is crucial in LOOC, where the goal is to image the sentinel nodes in the operating theatre within minutes of injection. Scatter and ‘shine-through effect’ in traditional tracers can impair immediate imaging, but Lymphoseek has not been compared with traditional tracers within the intraoperative setting. Over a longer protocol, Lymphoseek has shown impressive results in reducing the false negative rate for sentinel node biopsy (SNB in oral tumours from 9% to 2.56%. Due to licensing arrangements, Lymphoseek is only intermittently available in Europe; therefore, the LOOC trial pragmatically allows investigators the choice of Lymphoseek or a standard colloid tracer. This protocol allows for ad hoc interim analysis to ensure there is no significant difference in the total number of nodes and contralateral nodes identified by each tracer.

Review of the literature reveals a small number of case reports using freehand SPECT (fhSPECT) for intraoperative SNB. In one series, 23 patients with oral cancer had a 98% sentinel node detection rate by fhSPECT. Another series of 66 patients with oral cancer identified 94% of the sentinel nodes by fhSPECT. Drug posology, specifically radiation dose, is considered similar with Lymphoseek and 99mTc-human albumin colloidal particles. The research team has performed a blinded study comparing intraoperative sentinel node imaging fhSPECT with pre-operative SPECT/CT in 50 patients with oral cancer. SNB using fhSPECT alone was superior to SPECT/CT (false negative rate 5.3% vs 15.8%, respectively).^20^ Currently, there are no reports using Lymphoseek or fhSPECT in OPC, but the outlined data above are used as proof of concept.

## Methods and analysis

### Objectives

LOOC is a phase II surgical imaging trial for OPC. The overarching aim and objective is to establish the lymphatic drainage pattern and occult metastatic rate in the contralateral neck of patients with OPC.

**Figure 1:**
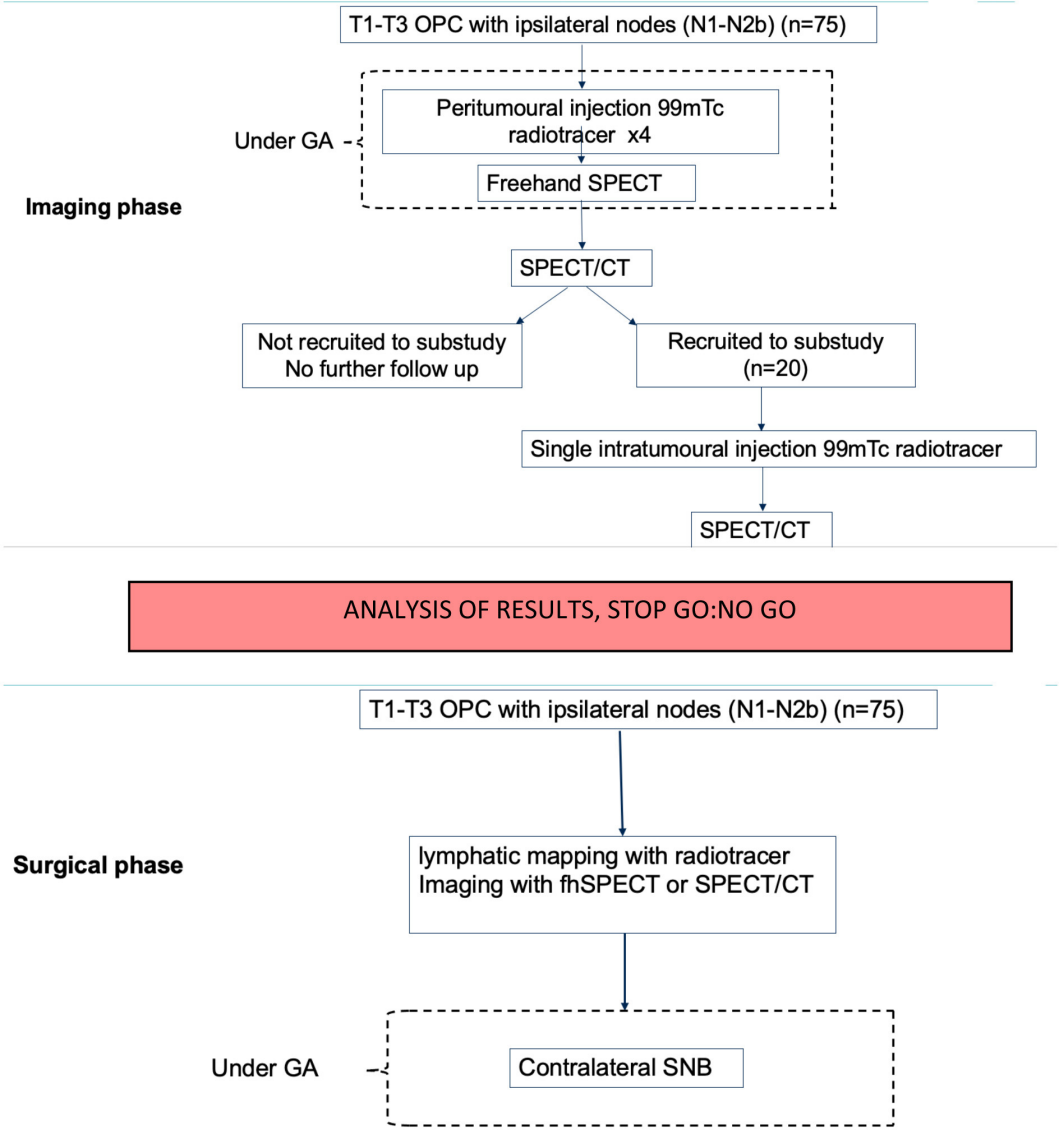
Trial design and patient care pathway of imaging phase (above) and surgical phase (below) with STOP GO:NO GO decision at trial mid-point. fhSPECT, freehand single photon emission CT; GA, general anaesthesia; OPC, oropharyngeal cancer; SNB, sentinel node biopsy.

The trial is divided into two phases—imaging and surgery (figure 1).

#### Imaging phase (n=75)

Objective: imaging protocol validation and establishing drainage pattern for patients with OPC with unilateral neck metastases.

##### Imaging substudy

Objective: to develop an alternative imaging protocol in case of failure of fhSPECT.

### Surgical phase (n=75)

Objective: feasibility of surgical staging of the contralateral neck in OPC with unilateral neck metastases.

### Trial design

LOOC is a multicentre prospective non-randomised phase II surgical imaging trial. This trial comprises two stages. Patients with newly diagnosed OPC with cervical metastasis will be considered for recruitment. The patient care pathway is shown in figure 1. The trial duration per participant is 4 weeks, with an estimated total trial duration of 54 months.

### Trial setting

The trial is being conducted in the secondary care hospital setting, within five high-volume UK head and neck (H&N) cancer sites—defined as a centre which sees over 100 new OPC diagnoses per year. Additional sites will act as participant identification centres in a ‘hub and spoke’ model, that is, smaller hospitals that refer into a tertiary cancer centre.

### Eligibility criteria

#### Inclusion criteria

- Adults aged 18 or over.
- New diagnosis of OPC—all anatomical subsites and HPV status accepted.
- Unilateral metastatic nodes equating to American Joint Committee on Cancer tumour, node, metastasis (TNM) 8th edition clinical staging N1-N2b for p16-negative patients, and N1 for p16-positive patients.

#### Exclusion criteria

- Suspicious bilateral nodes on imaging.
- Previous radiotherapy or surgery to the neck.
- Second primary oropharyngeal tumours.
- Distant metastasis (eg, lung, bone).
- Pregnancy and lactation.
- Inability to give informed consent.
- Allergy to lymphatic tracers.

### Planned interventions

The planned interventions in both the imaging and surgical phase of the LOOC trial are intended to occur opportunistically during the standard patient care pathway. When patients undergo an examination under anaesthesia (EUA) as part of surgical planning, or dental extractions under general anaesthesia (GA) prior to planned radiotherapy, the LOOC trial interventions will be conducted under the same GA procedure.

### Imaging phase

1. Four submucosal peritumoural injections of radiotracer are administered under direct/endoscopic vision during EUA.
2. After 15 min drainage, fhSPECT scan is undertaken (declispeSPECT, Surgiceye). Separate scans of both the contralateral and ipsilateral neck are taken.
3. Following recovery (and up to 24 hours later), conventional SPECT/CT is undertaken (gold standard imaging).

#### Imaging substudy

A subgroup of patients from the imaging phase (n=20) whose tumour is easily accessible without a GA (ie, clearly visible intra-orally) will be recruited to the imaging substudy. It is mandatory that patients recruited to the imaging substudy demonstrated nodal drainage on SPECT/CT (desirable if this was contralateral but not essential).

1. In the outpatient setting, 4–10 days after the initial imaging phase, a topical anaesthetic spray will be applied to the tumour, followed by a single injection of radiotracer placed directly into the tumour.
2. This will be followed by a SPECT/CT and a questionnaire assessing the acceptability of this outpatient-based procedure.

Due to interruptions in Lymphoseek tracer supply in the UK during the trial period, coupled with widespread staffing pressures on radio-pharmacy and nuclear medicine departments, a pragmatic decision has been made to add the choice of 99mTc-human albumin colloidal particles as one of the radiotracers allowed for this trial. There have been no high-quality head-to-head comparisons of 99mTc-human albumin colloidal particles and Lymphoseek in any tumour group, although small studies have shown that Lymphoseek may identify more sentinel nodes but without impact on the number of positive nodes identified.^21 22^ No studies report the sentinel node identification rate in 99mTc-human albumin colloidal particles or Lymphoseek under 1 hour post-injection. Thus, we will seek to compare sentinel node identification rate on fhSPECT in two groups of patients to ensure there is no gross disparity at this early stage of lymphatic mapping. If 99mTc-human albumin colloidal particles do not match Lymphoseek performance (sentinel node identification rate at up to 30 min postinjection), we will revert to Lymphoseek as the sole tracer.

#### Surgical phase

The surgical phase is planned to follow immediately from the imaging phase if specified outcomes are met. The precise surgical protocol is to be informed by outcomes of the prior imaging phase. Either:

Intraoperative peritumoural tracer injection and fhSPECT imaging of contralateral sentinel node in theatre under a single GA.

or

Single intratumoural injection in the outpatient setting, followed by conventional SPECT/CT.

Either of the above is followed by an SNB (as per standard protocol)^23^ of contralateral nodes during EUA. As per accepted evidence, the presence of viable individual tumour cells or greater is considered metastatic nodes.^24^ The result of the SNB will be discussed with the patient and the oncology team prior to further treatment, but the recommendation will be based on the decision of the multidisciplinary team (MDT). This trial is not powered to recommend changes in treatment based on the SNB result.

#### Subsequent assessments and procedures

The trial procedures are very safe, but all patients will be screened for adverse reactions following the final scan or the day following SNB and all complications will be reported according to the following protocol.

#### Definition of end of trial

The expected duration of the trial is 4 years from recruitment of the first participant. The end of the trial is the date of the last follow-up of the last participant.

The trial will be stopped prematurely if:

- This is mandated by the ethics committee.
- Following recommendations from the sponsor.
- Funding for the trial ceases.
- The Chief Investigator (CI), in consultation with the clinical and scientific lead, decides that sufficient biopsies and data have been obtained to fulfil the scientific objectives of the trial.

The Research Ethics Committee (REC) will be notified in writing within 15 days if the trial has been concluded or terminated early.

### Discontinuation/Withdrawal of participants

Participants will be free to withdraw from the trial at any time. No data or follow-up information will be collected in relation to participants from the date of withdrawal. The decision of a participant to withdraw from the trial will be recorded in the case report form (CRF). All recorded data and samples processed prior to the date of withdrawal of consent for trial participation will remain in the trial database and continue to be analysed as per trial protocol. In line with General Data Protection Regulations (GDPR), participants’ rights to access, change or move their information are limited, as these need to be managed in specific ways in order for the research to be reliable and accurate. On withdrawal from the trial, information already obtained will be kept, and no new data from the date of withdrawal will be held.

### Outcomes

#### Imaging phase

Primary outcome:

- Rate of contralateral drainage. fhSPECT and SPECT/CT images reviewed by two independent assessors. Sum of non-duplicated contralateral hotspots taken as the true contralateral drainage rate. Minimum of 20/75 patients must demonstrate contralateral drainage to proceed to surgical stage.

Secondary outcome:

- Accuracy of fhSPECT compared with SPECT/CT. Number of contralateral nodes on SPECT/CT used as denominator in calculating sensitivity of fhSPECT by independently verified images.

#### Surgical phase

- Occult metastatic rate of contralateral nodes (positive SNB).

### Participant timeline

A participant timeline and assessments conducted during the trial for the imaging and surgical phases are detailed in tables1^3^.

**Table 1:** Trial assessments performed for the imaging phase (stage 1) main group

Assessments	Screening	Baseline	Examination day	Examination day+24 hours
All stage 1 patients (n=75)				
Inclusion and exclusion criteria	x			
Consent	x			
Registration		x		
Demographic data		x		
Medical history		x		
Concomitant medications		x		
Tumour characteristics		x		
Examination under anaesthesia (standard of care)			x	
Injection of radiotracer and fhSPECT scanning			x	
Sentinel node imaging by SPECT/CT up to 24 hours postinjection				x
AE reporting			x	x

AE, adverse event; fhSPECT, free-hand single photon emission CT.

**Table 2:** Trial assessments performed for the imaging phase (stage 1) subgroup

Assessments	Screening	Baseline	Examination day	Examination day+24 hours	Examination day+4–10 days
Stage 1 subgroup (n=20)					
Inclusion and exclusion criteria	As per stage 1 main group				
Consent					
Registration					
Demographic data					
Medical history					
Concomitant medications					
Tumour characteristics					
Examination under anaesthesia (standard of care)					
Injection of radiotracer and fhSPECT scanning					
Sentinel node imaging by SPECT/CT up to 24 hours postinjection					
AE reporting					x
Local anaesthesia					x
Intratumoural injection of radiotracer					x
SPECT/CT scan					x
Patient acceptability questionnaire					x

AE, adverse event; fhSPECT, free-hand single photon emission CT.

**Table 3:** Trial assessments performed for the surgical phase (stage 2) main group

Assessments	Screening	Baseline	Scanning day*	Examination day
Stage 2 patients (n=75)				
Inclusion and exclusion criteria	x			
Consent	x			
Registration		x		
Demographic data		x		
Medical history		x		
Concomitant medications		x		
Tumour characteristics		x		
Outpatient radiotracer injection followed by SPECT/CT*			x*	
Examination under anaesthesia (standard of care)				x
Injection of radiotracer and fhSPECT scanning*				x*
Excision of contralateral nodes identified on imaging				x*
AE reporting			x	x

* scanning protocol dependent on outcome of imaging phase

AE, adverse event; fhSPECT, free-hand single photon emission CT.

### Sample size

Extrapolating from oral cancer models, our sample size is pragmatically based on the number of eligible cases seen and minimum cases required to establish a baseline lymphatic pattern.

### Imaging phase

For 75 patients our expected agreement is 96%, with an exact 95% CI width of approximately 10%. In addition, we will have 80% power to demonstrate that the agreement exceeds 88% using a one-sided test at a 5% significance level.

### Surgical phase

75 patients will be considered for SNB. Of these, 20%–30% (15–23 patients) are expected to have sentinel nodes on the opposite side of the neck. An exact 95% CI for this proportion is expected to have precision of ±11%.

### Recruitment

Patients diagnosed with OPC who fulfil the inclusion/exclusion criteria will be identified during H&N MDT meetings and in outpatient clinics. In each stage, up to 75 patients will be recruited over 18 months at five centres.

### Data collection methods

#### Pre-intervention assessments

No trial-specific procedures will be needed to be carried out to assess eligibility. All information required to determine eligibility will be available from the standard medical records and identified as specified above either during the H&N MDT meeting or in the outpatient clinic.

Relevant clinical information will be recorded in the CRFs. Data recorded will include:

- Demographics.
- Relevant medical history.
- Concomitant medication.
- Tumour characteristics, including size, type, grade, TNM stage, p16 status.

#### Participant registration

Consented, eligible participants will be registered by completing the trial’s online registration form. A unique pseudo-anonymised subject number will be generated. This number will be used to identify all patient data and tissue samples for the trial.

#### Data collection tools and source document identification

Data will be collected from sites using electronic CRFs (eCRFs). Source data are contained in source documents (medical records, which include laboratory and other clinical reports, case notes and hospital trust computer databases) and will be accurately transcribed onto the eCRF. The delegation log will identify all those personnel with responsibilities for data collection and handling, including those who have access to the trial database.

#### Completing case report forms

Data will be collected by eCRFs and will be verified using manual and electronic validation checks. All eCRFs will be completed by staff that are listed on the site staff delegation log and authorised by the Principal Investigator (PI) to perform this duty. The PI is responsible for the accuracy of all data reported in the eCRF.

#### Imaging phase outcome measure data collection

Each assessor fulfils a CRF per case with three questions:

1. Are there contralateral nodes on SPECT/CT? No/Yes (record neck level).
2. Are there contralateral nodes on fhSPECT? No/Yes (record neck level).
3. Do the contralateral nodes co-localise? Yes/No.

If contralateral nodes are shown in SPECT/CT but not on fhSPECT, this will be recorded as a false negative result (unless these can be attributed to second echelon nodes) for fhSPECT and vice versa. Contralateral drainage on either modality will be recorded as a true positive result. fhSPECT should achieve sensitivity >94% compared with SPECT/CT.

### Data management

A member of the local trial team will submit the data into the trial database. Access to the eCRF system will only be provided to staff with relevant authority delegated to them on the site’s delegation log. At enrolment, participants will be given a unique subject number and data will be entered under this subject number onto the trial database. No personal identifiable data will be stored on the trial database. Any personal identifiable data will be stored on a dedicated secure trial area, part of University College London’s (UCL) Data Safe Haven.

The trial is compliant with the requirements of General Data Protection Regulation (2016/679) and the Data Protection Act (2018). All investigators and trial site staff will comply with the requirements of the General Data Protection Regulation (2016/679) with regard to the collection, storage, processing and disclosure of personal information, and will uphold the Act’s core principles.

### Statistical methods

#### Validating image protocol

Patients will be assessed with both imaging procedures (fhSPECT and SPECT/CT), with SPECT/CT being considered the gold standard—to allow assessment of sensitivity and specificity of fhSPECT (including positive predictive value and negative predictive value). The agreement between fhSPECT and SPECT/CT will be recorded as either agree or disagree and will be calculated with an exact 95% CI. Two independent assessors will consider each pair of scans. We will investigate the agreement between these assessors and quantify the corresponding intra-class correlation.

#### Surgical phase

We will quantify the proportion of SNB patients with sentinel nodes on the opposite side of the neck with an exact 95% CI.

### Data monitoring

#### Trial Steering Committee and Data Monitoring Committee

A trial-specific Trial Steering Committee (TSC) and an independent Data Monitoring Committee have been appointed. The CI will report on scientific progress to these committees. Each of these committees will meet at least annually to monitor the progress of the trial and the safety of the trial, respectively.

#### Interim analyses

After the first five cases, a quality assurance analysis will be conducted by the data monitoring group to ensure adequate fhSPECT/SPECT-CT imaging quality for further analysis.

### Harms

#### Assessment and management of risk

Table 4 summarises the risks and mitigations of all interventions above standard care that are to be performed. The definition of adverse events (AEs) is provided in table 5.

**Table 4:** The risks and mitigations of all interventions above standard care

Intervention	Potential risk	Risk management
Radiotracer	Hypersensitivity (patients)	Ask patients about prior reactions to drugs, especially dextran or modified forms of dextran and exclude patients who are allergic. Observe for hypersensitivity signs and symptoms following radiotracer injection. Have resuscitation equipment and trained personnel immediately available.
Radiotracer	Adverse reactions (patients)	The most common adverse reactions (incidence <1%) are injection site irritation and/or pain. We will check for pain and administer analgesics to patients as required.
Radiotracer/SPECT	Radiation exposure (patients)	The effective dose equivalent of radiation exposure to an average dose used in a 70 kg adult is about 0.30 mSV (30 millirem) in males and is about 0.33 mSV (33 millirem) in females. Patients are also exposed to an additional 200 millirem of exposure to CT scan. Therefore, the total radiation exposure per scan is 230 millirem. Some patients (20/150 participants) will undergo two procedures. Therefore, such participants are exposed to an additional 460 millirem because of being involved in the project. The remaining 130/150 participants will be exposed to an additional 230 millirem because of being involved in the project. This is less than the average radiation exposure to a single CT chest. The average radiation exposure per year per individual due to background radiation is about 310 millirem. Therefore, the effective dose equivalent due to participation in the project is about 460/310 years or about 18 months of background radiation. To put this in context, the average radiation in a flight journey of about 1 hour at an altitude of 39 000 ft is about 0.006 mSV (0.6 millirem). Therefore, the dose received is equivalent to 460/0.6=767 hours of air travel.
Radiotracer/fhSPECT	Additional operation time	Total procedure including scanning adds 15–30 min to anaesthetic time. In the case of slow drainage after intraoperative injection, a second scan can be taken 10–15 min after the initial scan. The participants included in the study are those who are fit for major surgery. Therefore, the addition of the 15–30 min of time will not increase the risk significantly.
Sentinel node biopsy	Additional surgical procedure under GA and related wound complications including infection, bleeding, lymph collections	Qualified and trained doctors (surgeons and anaesthetists) will perform the procedure to minimise the risk due to GA and wound complications related to the biopsy.
Radiotracer/SPECT	Radiation exposure (healthcare professionals)	(1) Use waterproof gloves, effective radiation shielding and appropriate safety measures when preparing and handling radiotracer. (2) Radiotracer will be used by or under the control of physicians who are qualified by specific training and experience in the safe use and handling of radionuclides, and who have received approved training. (3) CT scan will be performed by trained healthcare professionals, who are aware of the radiation exposure to CT scan and take adequate precautions to minimise their exposure.

fhSPECT, free-hand single photon emission CT.

**Table 5:** Definitions of AEs

Term	Definition
AE	Any untoward medical occurrence in a patient or study participant, which does not necessarily have a causal relationship with the procedure involved.
Serious AE	Any AE that: Results in death. Is life-threatening.* Requires hospitalisation or prolongation of existing hospitalisation.† Results in persistent or significant disability or incapacity, or consists of a congenital anomaly or birth defect

Hospitalisation for pre-existing conditions, including elective procedures do not constitute an SAE.

* A life-threatening event refers to an incident in which the participant was at risk of death at the time it occurred; it does not refer to a hypothetical situation in which the event might have caused death had it been more severe.

† Hospitalisation is defined as an inpatient admission, regardless of length of stay.

AE, adverse event; SAE, severe adverse event.

The participants are not anticipated to have any unexpected AEs in this trial. The period of observation for events is until the following day of the procedure. AEs and SAEs will be recorded until the end of the period of observation.

It is not required to report expected AEs, and an AE form does not need to be completed for an expected AE. Expected AEs do not need to be reported to the sponsor.

A list of expected AEs includes the following:

- Injection site irritation.
- Pain.
- Allergy to radiotracer.

If any of these symptoms are accompanied by events consistent with the definition of an SAE as specified in table 5, then the event will be considered an SAE.

National Cancer Imaging Translational Accelerator (NCITA) will be informed of any SAE within 24 hours of the investigator becoming aware. Unexpected, related SAEs should be reported to NCITA within 24 hours of the investigator becoming aware. This will then be escalated to the sponsor.

Local site Research & Development (R&D) protocols for reporting SAEs will also be followed. All SAEs will be followed up until a resolution is reached (ie, recovered, recovering, recovered with sequelae, fatal, not recovered or unknown).

#### Assessments of adverse events

Each AE will be assessed for severity, causality, seriousness and expectedness as described below.

#### Causality

The assessment of the relationship of AEs to the procedure is a clinical decision based on all available information at the time of the completion of the CRF.

The following categories listed in table 6 will be used to define the causality of the AE.

**Table 6:** Categories for causality of the adverse event

Category	Definition
Definitely	There is clear evidence to suggest a causal relationship, and other possible contributing factors can be ruled out.
Probably	There is evidence to suggest a causal relationship, and the influence of other factors is unlikely.
Possibly	There is some evidence to suggest a causal relationship (eg, the event occurred within a reasonable time after administration of the study procedure). However, the influence of other factors may have contributed to the event (eg, the participant’s clinical condition, other concomitant events).
Unlikely	There is little evidence to suggest there is a causal relationship (eg, the event did not occur within a reasonable time after administration of the study procedure). There is another reasonable explanation for the event (eg, the participant’s clinical condition).
Not related	There is no evidence of any causal relationship.
Not assessable	Unable to assess on information available.

#### Expectedness

The expected is defined as shown in table 7.

**Table 7:** Expectedness of adverse event

Category	Definition
Expected	An adverse event that is consistent with the information about the procedure defined in this protocol.
Unexpected	An adverse event that is not consistent with the information about the procedure defined in this protocol.

#### Recording adverse events

All AEs that occur during the period of observation (which is the next day after the procedure) should be recorded on the AE log. Expected and related events do not need to be reported.

#### Recording and reporting serious adverse events

All reportable serious AEs will be recorded in the medical records and the appropriate eCRF and the AE log.

### Auditing

#### Trial Management Group

The trial management group (TMG) will include the CI, the clinical and scientific leads for the trial and the NCITA operational team. The TMG will be responsible for overseeing the trial. The group will meet at least every 6 months during the period of recruitment and annually during the follow-up period and will send updates to PIs. The TMG will review recruitment figures, the ongoing progress of the scientific studies and any resulting necessity for modification of the characteristics of subjects to be recruited to the trial and any consequent requirement for substantial amendments to the protocol prior to submission to the REC. All PIs will be kept informed of substantial amendments. The TMG will additionally submit periodic progress reports to the REC and sponsor.

#### Trial Steering Committee

A trial-specific TSC will be appointed. The CI will report on scientific progress to this committee, which will meet at least annually to monitor both the progress and safety of the trial.

Before any NHS site may be opened to recruit participants, the CI/PI or designee must receive NHS permission in writing from the Trust R&D. It is the responsibility of the CI/PI or designee at each site to ensure that all subsequent amendments gain the necessary approvals, including NHS permission (where required) at the site. This does not affect the individual clinician’s responsibility to take immediate action if deemed necessary to protect the health and interests of individual participants.

An annual progress report (APR) will be submitted to the REC within 30 days of the anniversary date on which the favourable opinion was given, and annually until the trial is declared ended. The CI will prepare the APR. Within 90 days after the end of the trial, the CI/sponsor will ensure that the main REC is notified that the trial has finished. If the trial is terminated prematurely, those reports will be made within 15 days after the end of the trial.

The CI will supply the sponsor with a summary report of the trial, which will then be submitted to the REC within 1 year after the end of the trial.

## Ethics and dissemination

### Research ethics approval

The trial protocol, participant information sheet, consent form, GP letter and other supporting documents have been approved by the Health Research Authority and the Yorkshire & The Humber-South Yorkshire Research Ethics Committee (REC ref: 20/YH/0111). The LOOC trial is registered with ClinicalTrials.gov, ID: NCT04498221.

### Protocol amendments

Protocol modifications will be notified to the competent authority, ethics committee and investigators by the LOOC trial office.

### Consent

Patients who fulfil the eligibility criteria will be provided with a patient information sheet (PIS) by the investigator or a designated appropriately trained member of the research team, who will be present to answer any questions regarding the aims, methods, anticipated benefits and potential hazards of the trial. They will explain that participants are under no obligation to enter the trial and that they can withdraw at any time during the trial, without having to give a reason.

The person taking consent will be suitably qualified and experienced and will have been delegated this duty by the PI on the Staff Signature and Delegation of Tasks log. Potential participants will be offered sufficient time (at least 24 hours) to consider the trial, allowing time for discussion with family/friends/GP. The participant will be given the opportunity to ask questions and to be satisfied with the responses prior to written consent being taken. No trial procedures will be conducted prior to the participant signing the trial consent form. Following consent, the patient will be enrolled in the trial and allocated a unique pseudo-anonymised subject number. A copy of the signed informed consent form will be given to the participant. The original signed form will be retained in the investigator site file and a copy placed in the medical notes. The PIS and consent form will be reviewed and updated if necessary throughout the trial (eg, where new information becomes available) and participants will be re-consented as appropriate.

### Confidentiality

All personal identifiable data collected during the trial will be handled and stored in accordance with the Data Protection Act (2018) and GDPR and all other applicable regulations and legislation. To preserve patient anonymity, only the allocated subject number and subject identifier will be recorded on the CRFs. Medical records (case notes and hospital trust computer databases) may be accessed by the recruiting site for up to 4 years from the date of consent for the purpose of data clarification. Information about participant demographics, medical history and concomitant medication and tumour characteristics, together with clinical follow-up information, will be made available to the trial team.

### Access to data

#### Archiving

UCL and each participating site recognise that there is an obligation to archive trial-related documents at the end of the trial. The CI will archive the trial master file at UCL and the PI at each respective site will locally archive trial documents for 20 years and in line with all relevant legal and statutory requirements.

#### Intellectual property

No background intellectual property rights (including licences) are required and no commercially exploitable intellectual property is likely to be generated during this research.

#### Data sharing statement

Once the initial trial publication has been released, all participant data collected will be de-identified and shared as per the NCITA policy, which allows access via request to the TMG. Once all trial publications (primary, secondary, tertiary) are in the public domain, the data will be open access. The study protocol, statistical analysis plan and informed consent form will also be available through planned open access publication.

#### Ancillary and post-trial care

UCL holds insurance against claims from participants for injury caused by their participation in the trial. Participants may be able to claim compensation if they can prove that UCL has been negligent. However, as this trial is being carried out in a hospital, the hospital continues to have a duty of care to the participant of the trial. University College London does not accept liability for any breach in the hospital’s duty of care, or any negligence on the part of hospital employees. This applies whether the hospital is an NHS Trust or otherwise.

Participants may also be able to claim compensation for injury caused by participation in this trial without the need to prove negligence on the part of UCL or another party. Participants who sustain injury and wish to make a claim for compensation should do so in writing in the first instance to the CI, who will pass the claim to the sponsor’s Insurers, via the sponsor’s office.

Hospitals selected to participate in this trial shall provide negligence insurance cover for harm caused by their employees and a copy of the relevant insurance policy or summary shall be provided to UCL, on request.

### Dissemination policy

The results of this research will be published in academic journals. Authorship will reflect the individual contribution to research in line with standard academic practice. Results will be disseminated to patients and the public via local/national patient groups, cancer charities and social media/press releases. The contribution of the funders of this research and the clinicians contributing to the research will be acknowledged.

### Patient and public involvement

The UCL Head & Neck Academic Centre patient group was involved in the design of this study protocol. The patient group approved the PIS and substudy patient questionnaire. The patient group and study patient representative will be involved in the dissemination of results.

#### Trial status

As of the publication date, the LOOC trial is actively recruiting participants. Recruitment began in July 2022 and is expected to conclude in January 2027.
