# Supplementary material for: Lymphatic mapping Of Oropharyngeal Cancer (LOOC): protocol for a phase II surgical imaging trial to evaluate contralateral drainage and occult metastasis in oropharyngeal cancer
Source: BMJ Open. 2025 Jun 5;15(6):e101746. doi: 10.1136/bmjopen-2025-101746 (PMC12142040; doi:10.1136/bmjopen-2025-101746)
Supplement: Supplementary file 1 [file bmjopen-2025-101746.reviewer_comments.pdf]

PEER REVIEW HISTORY

BMJ Open publishes all reviews undertaken for accepted manuscripts. Reviewers are asked to complete a checklist review form and are provided with free text boxes to elaborate on their assessment. These free text comments are reproduced below.

ARTICLE DETAILS

Title (Provisional)

Lymphatic mapping Of Oropharyngeal Cancer (LOOC): protocol for a phase II surgical imaging trial to evaluate contralateral drainage and occult metastasis in oropharyngeal cancer

Authors

Schilling, Clare; Payne, Karl; Wan, Simon; Brew-Graves, Chris; Ofo, Enyi; Ambler, Gareth; Weller, Alex; Gnanasegaran, Gopinath; Paleri, Vinidh; Shaw, R; Fleming, Jason; Walker, David; Bajwa, Mandeep; McGurk, Mark

---

This article was not externally reviewed at BMJ Open. Protocols that have been independently assessed prior to submission to BMJ Open are usually fast-tracked to publication on the grounds that further substantial changes will not be possible. This independent assessment will usually be external, independent review for both a substantial grant award from a non-commercial or government funder and ethics approval.
